# The impact of flipped classroom on English proficiency of first-year Chinese urban and rural pre-service teachers

**DOI:** 10.3389/fpsyg.2024.1347826

**Published:** 2024-05-16

**Authors:** Wei Chen

**Affiliations:** School of Foreign Languages, Hainan Normal University, Haikou, China

**Keywords:** flipped classroom, English language teaching, pre-service teachers, English proficiency, educational background

## Abstract

**Introduction:**

In recent years, China has made strides in adopting student-oriented teaching approaches, particularly in tertiary English education, through the integration of enhanced technology. This study aimed to investigate the impact of flipped classroom on the English proficiency of first-year pre-service teachers at a Chinese normal university. It also sought to determine whether educational background (urban or rural) interacted with the teaching approach (flipped or traditional) in affecting the language proficiency of the learners.

**Methods:**

A quasi-experimental design was utilized with two treatments: a flipped classroom approach and a traditional teacher-centered teaching approach. Both approaches were implemented in the Integrated English Course over a 12-week semester. Two randomly selected classes, consisting of 60 pre-service teachers in each class, were assigned to either the experimental or control group. Data were collected from pretest and post-test assessments and analyzed using two-way ANOVA.

**Results:**

The results revealed a positive impact of the flipped classroom and a significant interaction between educational background and teaching approach on English proficiency. Specifically, urban pre-service teachers achieved higher English proficiency than their rural peers when taught in the flipped classroom, but not in the traditional classroom. Furthermore, urban pre-service teachers in the flipped classroom outperformed their urban peers in the traditional classroom, while rural pre-service teachers did not show any significant difference in their performance between the two classes.

**Discussion:**

The findings suggested that the flipped classroom approach was more effective than the traditional approach for Chinese pre-service teachers, particularly those with an urban educational background. However, it is important to ensure that rural learners receive sufficient support to benefit equally from this innovative teaching approach. Accordingly, implications and recommendations for future research are discussed.

## 1 Introduction

Learning English as a foreign language (EFL) is a challenging task for students in a dominant first language setting (Abdullah et al., 2019). In China, tertiary EFL learners are confronted with several difficulties that hamper their language achievements (Xiu, 2021). Among them, the traditional teaching approach, which prioritizes teacher-centered instruction and content regurgitation, is often criticized for contributing to the difficulties encountered by Chinese learners (Wang, 2020). This conventional approach is still commonly employed by language educators in Chinese universities, impeding students’ acquisition of English knowledge, skills, and associations they develop with their English learning (Yang and Chen, 2020). Moreover, educational backgrounds can significantly impact upon Chinese EFL learners. Due to conspicuous regional disparities in development between urban and rural areas of China (Wu et al., 2021), students from rural areas face more challenges in EFL learning than their urban peers (Liu and Wang, 2021). Hence, supporting Chinese students of diverse educational backgrounds with potentially more effective teaching approaches seems to be needed.

In the last decade, the advert of technology and its application have brought about tremendous changes in EFL teaching and learning (Haghi, 2021). Quite a few language educators worldwide have adopted gradual steps to integrate technologies into their teaching approaches (Basal, 2015). As an active-learning, student-centered, and flexible pedagogical approach incorporating digital technology, flipped classroom has gained growing prominence, especially at the tertiary level (Kurt, 2017). Its increasing popularity is attributed to research suggesting that flipped classroom may contribute to positive language learning outcomes (Sukerti et al., 2020; Gok et al., 2021; Pang, 2022; Liu et al., 2023). Nevertheless, critical evaluations concerning its effectiveness and contextual applicability has also been raised (Alhamami and Costello, 2019; Lakarnchua et al., 2020; Altas and Enisa, 2021; Rajabi et al., 2021).

Currently, the implementation of flipped classroom in EFL education in China has revolutionized the traditional teaching model and invigorated modern classroom teaching (Yang and Chen, 2020). In response to the Chinese higher education informatization reform, an increasing number of front-line teachers and researchers are adopting the flipped classroom approach to promote English learning (Wang et al., 2021). While several studies have identified the benefits of flipped EFL classroom in the Chinese context, its effectiveness is still subject to debate (Zhang and Guo, 2018; Chen, 2019). Some critics argue that this approach may lead to unequal language achievements among urban and rural students, emphasizing the need to consider students’ educational background as a context-specific factor (Lv and Wang, 2016). So far, the use of flipped classroom in EFL teacher education in China is still under researched. Therefore, this study aimed to fill the gap by investigating the implementation and impact of flipped classroom on English proficiency of Chinese pre-service teachers while considering the potential effect of their educational backgrounds. It answered the following the research questions.

1) Is there a significant difference in English proficiency between first-year Chinese pre-service teachers exposed to the flipped classroom and those engaged in the traditional teacher-centered instruction?
2) Does educational background (urban or rural) significantly interact with teaching approach (flipped or traditional) in influencing English proficiency among the pre-service teachers?

Accordingly, two null hypotheses were tested.

*H01*: There is no significant difference in English proficiency between first-year Chinese pre-service teachers exposed to the flipped classroom and those engaged in the traditional teacher-centered instruction.

*H02*: There is no significant interaction between educational background and teaching approach in terms of English proficiency of the pre-service teachers.

## 2 Literature review

### 2.1 EFL learning and influencing factors

EFL learning refers to the systematic acquisition of English language proficiency through formal instruction, typically in a language classroom setting. In many contexts where English is not the dominant native language, such as Russia, Egypt, Thailand, Japan, and China, EFL learners have minimal exposure to English outside of the classroom (Jenkins, 2003). As a result, they face significant challenges in achieving proficiency in English. To overcome these challenges, it is crucial for both educators and learners to recognize and understand potential factors influencing EFL learning. Research has pinpointed a range of internal factors, spanning personality, motivation, cognitive style, attitude, self-confidence, anxiety, and previous educational backgrounds (Pariyanto and Pradipta, 2020). Furthermore, external factors, including teaching approaches, learning materials, environment, and social elements such as peer groups, play a pivotal role in shaping the EFL learning experience (Getie, 2020). While these factors appear intricately intertwined and jointly impactful in EFL learning, some researchers emphasize the significance of specific learner and teacher factors, such as previous educational background and teaching approach (Cuong, 2021; Ömer and Akçayoğlu, 2021). This study, therefore, sought to investigate the possible influences of these factors on the EFL learning of pre-service teachers in China and propose implications accordingly.

### 2.2 Educational background and urban-rural disparities in EFL education

Educational background encompasses both formal and informal education, as well as any ongoing learning pursuits (Glassdoor Team, 2021). It not only reflects geographic features, but also embodies historical, cultural, and educational characteristics of a local area, exerting an impact on individual subjects (Evans and Savage, 2015). In this study, educational background, as a contextually specific factor, was defined as an urban or rural area where a student grew up and attended his/her secondary school, categorized according to China’s national statistics on urban and rural districts (National Bureau of Statistics, 2020).

Previous research has revealed that EFL learners with rural educational backgrounds tend to possess lower linguistic attainment, inadequate learning motivations, and higher language anxiety compared to their urban peers (Cuong, 2021). To address these disparities, some researchers argue that language teachers should identify students’ educational backgrounds and modify teaching methodologies accordingly (Deepa, 2021), while also leveraging technology as an educational equalizer (Li et al., 2018).

In China, urban–rural inequality in education has been a persistent problem across all levels of education (Rural Education Action Program, 2018; Wu and Zhang, 2021), primarily resulting from the structural duality in social and economic systems (Feng et al., 2019). In recent years, the Chinese government has made significant efforts in scaling up education in rural areas (Yue et al., 2018), reflecting its commitment to achieving educational equality. In furtherance of this goal, the Communist Party of China Central Committee and The State Council (2018) released the National Rural Vitalization Strategic Plan (2018–2022), with a focus on promoting education in rural areas. Moreover, the Chinese Ministry of Education (2012) issued “Plan for ICT in Education 2011–2020,” advocating adoption of information technology and technology-assisted teaching approaches. Despite progress in alignment with these policies, disparities persist, particularly in the domain of EFL education (Liu and Wang, 2021). Previous studies have shown that rural students achieve significantly lower English scores in standardized tests compared to their urban peers (Ke, 2016), and face challenges and problems in communication (Liu, 2021), foreign language anxiety (Chen, 2020), classroom participation (Ou, 2017), learning motivation (Liu and Wang, 2021), digital literacy, and use of cognitive, meta-cognitive and emotional regulation strategies (Zhang, 2022). It seems that much remains to be done to bridge the regional gaps in EFL education and the implementation of innovative teaching approaches tailored to the specific needs of Chinese EFL learners from different educational backgrounds requires further exploration.

### 2.3 Flipped classroom

Flipped classroom was first defined by Lage et al. (2000) as an instructional approach in which “events that have traditionally taken place inside the classroom now take place outside the classroom and vice versa” (pp. 30–43). This broad definition is a starting point to provide teachers freedom and flexibility to adopt the flipped classroom with various methods (Eppard and Rochdi, 2017). However, the broad definition is later on restricted by some researchers who limit the concept of flipped classroom by inclusion of specific methods like instructional videos outside of class as well as interactive group learning inside of the class (Bishop and Verleger, 2013). The narrow definition suggests that information transmission occurs out of the classroom, while the assimilation takes place in the classroom (Talbert, 2012).

The flipped classroom is a promising instructional approach with theoretical underpinnings in both cognitivism and constructivism. Cognitivism supports the flipped classroom through a process that involves students in lower levels of cognitive work prior to class, such as recalling and understanding concepts, and engages them in higher levels of cognitive activities to apply, analyze, synthesize, and evaluate during in-class time (Bloom, 1984). The revised Bloom’s Taxonomy is even more relevant to flipped classroom, as it shows a dynamic cognitive process from information transmission, occurring independently outside of class, to information assimilation, requiring more critical reasoning under the guidance of a teacher during class and after class (Talbert, 2012). Constructivist paradigms, including cognitive and social constructivism, are also key theoretical foundations for the flipped classroom. Teachers in flipped classes need to adjust students’ learning process with their cognitive stages, facilitate problem-solving skills in an active learning environment, and encourage collaborative tasks that help students construct knowledge through social interaction. In constructivist flipped classes, learners become active participants and information constructors, building up their subjective representations of knowledge based on prior experience (Bergmann and Sams, 2012a). To facilitate students’ knowledge construction, teachers should organize interactive discussions, create active learning environments based on questioning, and offer immediate feedback and differentiated support to scaffold students’ flipped learning.

### 2.4 Impact of flipped classroom on English proficiency

Flipped classroom is a teaching approach that utilizes digital technologies to facilitate independent learning outside of the classroom, and interactive learning activities inside the classroom (Bergmann and Sams, 2012b). This technology-assisted teaching approach has been reported to produce a number of positive outcomes in EFL learning at various levels (Strelan et al., 2020), such as improved language proficiency (Sukerti et al., 2020), ameliorated foreign language anxiety (Gok et al., 2021), promoted critical thinking skills (Pang, 2022), boosted self-confidence, motivation and autonomy (Ghufron and Nurdianingsih, 2019), and enhanced learner engagement and satisfaction (Wu and Zhang, 2021). Among these positive outcomes, improved English proficiency has been underlined by many researchers (Roth and Suppasetseree, 2016; Loucky, 2017; Kırmızıa and Kömeç, 2019; Sukerti et al., 2020). For example, Kırmızıa and Kömeç (2019) found that Turkish high school students who engaged in flipped learning achieved significantly higher scores in English vocabulary quizzes than those taught by the traditional method. Similarly, Zhang et al. (2016) discovered that first-year English majors in a Chinese university were able to produce more language output, actively solved their own problems in a more engaging way, and increased their interest in language learning through the use of the flipped classroom approach.

However, the effectiveness of the flipped classroom on improving English proficiency is still open to debate. Some studies have found no significant difference in language proficiency of students taught by flipped and traditional teaching approaches (Suranakkharin, 2017; Yang, 2017; Alhamami and Khan, 2019), and there are also concerns about the potential impact of educational backgrounds on the effectiveness of this model (Lv and Wang, 2016; Liu and Wang, 2021). For instance, Lv and Wang (2016) found that rural Chinese students with less digital skills had higher levels of language anxiety and obtained lower scores in flipped classes compared to their urban peers. Therefore, while the flipped classroom shows promise in improving EFL education outcomes, further research is needed to explore its effectiveness in different contexts, taking into account the influence of cultural and educational backgrounds on language learning outcomes.

Despite the growing interest in implementing the flipped classroom in EFL classes, limited research has been conducted on its effectiveness in EFL teacher education, and there have been mixed results (García-Sánchez and Santos-Espino, 2017; Karaaslan and Çelebi, 2017). One study by García-Sánchez and Santos-Espino (2017) found that the flipped classroom approach empowered Spanish pre-service EFL teachers to improve their academic performance, while other studies by Adnan (2017) and Cabi (2018) found no significant difference in English learning outcomes between flipped and non-flipped classes. It seems that more research conducted in different contexts is needed to clarify the effectiveness of the flipped classroom on pre-service EFL teachers. Hence, this study may add to existing literature by providing valuable empirical evidence and instructive insights into the feasibility of this teaching approach in a specific Chinese context.

## 3 Methodology

### 3.1 Course context

The utilization of the flipped classroom was examined in Integrated English Course (IEC), which is a mandatory course for undergraduate English majors in China. According to the Teaching Guide for Undergraduate English Majors issued by the Ministry of Education of China in 2020, IEC is not solely a language course developed to equip students with basic English knowledge and skills to enhance their language proficiency (Jiang, 2019), but also a competence-building course aimed at promoting students’ lifelong learning skills such as problem-solving, communication, and collaboration (Qu, 2017; Wen, 2019). This course is offered to undergraduate English majors for the first two years (semester 1–4) of their university study. There are two classes per week, each lasting one and a half hours.

### 3.2 Research design

This research utilized a quasi-experiment with a 2 × 2 non-equivalent control group design to investigate whether the flipped classroom approach or traditional teacher-centered teaching approach had a significant impact on the English proficiency of first-year Chinese pre-service teachers with urban and rural educational backgrounds.

### 3.3 Participants

Participants involved in the study were 120 students from the Faculty of English Language Teaching at a normal university in China. All the participants were pre-service teachers in their first year of undergraduate studies and attending Integrated English Course over a 12-week semester in academic year 2022.

Before enrollment, these students, aged from 18 to 20, had learned English for at least 9 years in urban or rural schools, and most of them had no prior flipped classroom experience. They were randomly picked from nine classes and assigned to an experimental group and a control group. Each group comprised 60 pre-service EFL teachers (30 urban and 30 rural students). The male-to-female student ratio was approximately 1:3 in every group.

First-year pre-service teachers were chosen as the target participants due to their possible need of guidance and help as freshmen in their university study as well as positive outcomes the flipped classroom might bring to the cohort in their subsequent tertiary EFL learning and in their future career as EFL teachers.

Two carefully selected instructors were invited to teach in the experimental and control groups. Both were females, close in age and in working years, and similar in their educational backgrounds. The instructor assigned to the flipped classroom had four-year experience with it. Despite this, she still underwent a training session to familiarize with specific procedures required for the flipped course instruction.

### 3.4 Intervention

Two teaching approaches were implemented by the two language teachers, respectively. The control group received traditional teacher-centered teaching, which was the typical style of teaching in the EFL classroom. This approach focused more on the teacher than on the students, and the majority of class time was spent helping students remember and understand key concepts. In contrast, the experimental group was taught using the flipped classroom approach, which emphasized the importance of students being active in their technology-assisted learning supported and guided by the teacher.

In the traditional classroom, the teaching process followed the normal convention. Before class, students were asked to review textbook materials while the instructor prepared PowerPoint presentations. In class, new topics were explained in detail using lecture slides, supplemented with exercises to enhance comprehension. Occasionally, students were invited to share their work and opinions. At the end of class, the instructor summarized the topic, assigned homework, and shared additional materials online for self-study after class. If needed, students could seek assistance from the instructor outside of class if needed.

Common activities performed in the control group included teacher-led lectures, fast-reading, pair-work, group discussions and presentations. Lectures, consuming a a substantial amount of class time, utilized multimedia tools for content delivery. Fast-reading involved independent skimming, scanning, pair discussions, and class sharing. Group discussions and presentations emphasized collaboration, requiring students to collectively apply knowledge. These activities targeted integrated language skills, focusing on the cognitive domain. Due to lecture-focused time constraints, higher-order thinking activities were often conducted after class.

The flipped classroom was implemented in the following steps. Before each class, students were tasked with independently mastering key concepts at their own pace and completing a pre-class quiz. They could also post questions on an online forum within a Learning Management System (LMS) or via WeChat or QQ online study groups. The teacher then collated students’ results, selected key questions to be addressed during class, and designed relevant activities to help students apply what they had learned. The face-to-face class began with a brief lecture, followed by feedback from students using a voting system in the LMS. Individual and group activities were then conducted, with the teacher monitoring performance and providing guidance as needed. Both types of activities aimed to promote positive interactions among students and with the teacher. At the end of class, the teacher summarized the topic and unresolved issues, and students reflected on their learning and provided suggestions for future activities. After class, students continued to apply their knowledge by completing further exercises and engaging in discussions on the topic. The teacher offered ongoing guidance and adjusted lesson designs based on student feedback.

In the flipped classroom, a variety of individual activities (such as polling, individual problem-solving, brainstorming, crowd-sourcing, and individual presentations) and group-based activities (such as three-step-interviews, cumulative brainstorming, think-pair-share, and group presentations) were carefully designed and carried out to enhance learning outcomes progressively. For instance, the three-step-interview required students to work in pairs, interviewing each other about their gains and questions, encouraging independent or collaborative information seeking and problem-solving. This relatively low-difficulty activity aimed to help them remember and gain a better understanding of specific learning content. Following that, the think-pair-share with a certain level of complexity could be conducted to promote higher-level cognitive development. This activity required students to work independently with guidance from the instructor or collaboratively with their peers to gather and synthesize key points, create a concept map, and present their work in class. The activity not only aimed to help students better organize their knowledge and illustrate complex conceptual associations but also intended to improve their language skills, enhance their communication abilities, spark their interest in learning, and foster positive interpersonal relationships among classmates. Undoubtedly, various other activity options were utilized in the flipped classroom. These activities, used individually or in combination for each lesson, were tailored to the expectations and evolving needs of the pre-service teachers in the specific context.

In the experimental group, various technological tools were adopted to facilitate the flipped teaching and learning process. Common hardware like laptops, tablets, and smartphones supported the entire process, while public computers in the library were available for pre-service teachers with limited device access. Multimedia facilities, including interactive whiteboards, projectors, computers, and video recorders, were used for face-to-face instruction. During the preparation phase, software such as search engines, video editing tools, and text editing tools was employed. The LMS played a central role in content delivery, progress monitoring, and communication. Additionally, social media apps like WeChat and QQ complemented the LMS, serving as alternative platforms for material sharing, pre-class discussions, and feedback collection. These tools were strategically utilized throughout the in-class and post-class phases, effectively guiding the flipped teaching and learning.

For ethical considerations, teaching content for both groups was identical, and all resources provided to the experimental group were made available to the control group after the end of the study. Furthermore, the same intervention was implemented with the control group afterward.

### 3.5 Instrument and procedures

The instrument used in the study was two IELTS (International English Test System) tests selected from the IELTS Student’s Book published by Cambridge University (Cambridge, 2019). The two papers were modified and utilized as the pretest and the post-test.

The proficiency tests were chosen due to their widely recognized status for reliability and validity. The reliability, measured through Cronbach’s Alpha, averaged 0.88 for reading and listening modules (University of Cambridge Local Examinations Syndicate, 2007). While speaking and writing modules lacked reported reliability coefficients, their dependability was ensured through examiner training and standardization (Fazel and Ahmadi, 2011). The IELTS homepage attested to excellent internal consistency (coefficient: 0.95, SEM: 0.21) across all modules (Hashemi and Daneshfar, 2018). Past studies also affirmed predictive and construct validity, with IELTS tests predicting academic language performance (Ingram and Bayliss, 2007) and correlating positively with GPA (Yen and Kuzma, 2009). Despite construct validity requiring further exploration, satisfactory levels were reported across reading, listening, writing, and speaking modules, affirming precision of IELTS tests in reflecting language abilities (Quaid, 2018; Noor, 2020).

Moreover, the IELTS tests, specifically the General Training version, were selected because these tests closely aligned with the expected learning outcomes of the IEC in assessing language abilities. The adoption of the General Training version was based on its suitability for evaluating the language proficiency of participants, who were expected to enhance and demonstrate their English skills in practical daily contexts.

In terms of test components, each test paper consisted of four modules: listening, reading, writing, and speaking. The listening module included a recorded monolog and a daily conversation with 20 questions in total. This module measured students’ ability to understand opinions, purposes, and attitudes of speakers as well as ability to follow detailed information and idea development. The reading module contained two sections (20 questions) with factual texts on general topics. The reading module, focusing on real-world situations, evaluated students’ ability to understand main ideas, details, and implied meanings as well as ability to recognize authors’ arguments, attitudes, and purposes. The writing module was about a letter writing task requiring students to write at least 150 words. This part examined whether students were capable of engaging in personal correspondence to elicit information, express, and justify opinions, outline a problem, and present related solutions. And the speaking module, comprised of general questions about some familiar topics, a short presentation on a given topic, and a two-way discussion, assessed a wide range of speaking skills, including organizing ideas, justifying views, and analyzing and discussing issues. Each of the four test modules accounted for a quarter (25 points) of the total score (100 points). The first three modules, including listening, reading, and writing, lasted one and a half hours (30 min for each module) without a break. And the speaking module, given on the same day, lasted for 6–8 min for each student. The performance of students was evaluated by two examiners who had received standardized IELTS assessment training as scorers from a British Council’s IELTS test center in China. Using answer keys and band descriptors as reference points, these examiners provided scores for all four modules to all 120 participants. To further ensure the instrument’s validity and reliability, a pilot study was done to assess content validity, internal consistency, and inter-rater reliability of IELTS speaking and writing modules using analysis of intra-class correlation coefficients (ICC). A panel of five experts in English language education and assessment conducted content validation. They reviewed and scored each item, ensuring relevance and representation. Results showed high agreement (95%), confirming content validity. For internal consistency, another group of first-year pre-service teachers (*n* = 38) took an IELTS test, yielding a Cronbach’s alpha of 0.702, indicating acceptable consistency. Inter-rater reliability for subjective scoring in speaking and writing modules was assessed, resulting in excellent ICC values of 0.92 and 0.98, meeting established guidelines (Koo and Li, 2016). This comprehensive analysis ensured the instrument’s reliability and validity.

Before the intervention, a pretest was administered to both groups. Participants in the experimental group underwent a one-week training session, introducing them to components, requirements, procedures, activities, and potential benefits of the flipped classroom, given their lack of previous experience in this approach. Subsequently, they engaged in a two-week practice of the flipped classroom to become familiar with expectations both in and out of class. Following this, both groups were taught the same content from the English course, and the intervention lasted for 8 weeks. At the end of the treatment, a post-test was given to both groups. Test papers were collected, and data were analyzed to compare the English proficiency of the pre-service teachers in the groups. Moreover, during the pretest and post-test, demographic information about students’ class and educational background was collected for subsequent data analysis.

## 4 Results

All the data were analyzed quantitatively using the Statistical Packages for the Social Science (version 26).

First, a test of homogeneity of variances was conducted to examine whether all the subgroups were homogeneous in terms of their English proficiency at the beginning of the intervention. The assumption of homogeneity across all levels of the independent variables was met, indicated by the Levene’s test of equality of error variances, *F*(3, 116) = 2.61, *p* = 0.055 for the IELTS pretest score.

After confirmation of homogeneity of groups, assumption testing of the two-way ANOVA was followed. An inspection of the assumption of univariate normality demonstrated that the dependent variable was normally distributed for all the sub-groups (*ps* > 0.05), supporting the assumption of normality. There was just one mild univariate outlier and no extreme outliers for the dependent variable indicated by the boxplots. The assumption of homogeneity of variances was tenable, indicated by the Levene’s test of equality of error variances, *F*(3, 116) = 1.87, *p* = 0.138 for the scores. As all the assumptions were met, an analysis was conducted.

Table 1 reports the descriptive statistics of the IELTS post-test scores of the flipped classroom (FC) and traditional teacher-centered teaching classroom (TT), including the mean, standard deviation of values for each combination of the groups of the independent variables, as well as the number of participants for each cell.

**Table 1:** Descriptive statistics.

Teaching approach (TA)	Educational background (EB)	Mean	Std. deviation	*N*
TT	Rural	57.90	7.81	30
	Urban	58.30	7.61	30
	Total	58.10	7.65	60
FC	Rural	60.72	5.32	30
	Urban	68.88	6.00	30
	Total	64.80	6.97	60
Total	Rural	59.31	6.77	60
	Urban	63.59	8.64	60
	Total	61.45	8.02	120

The results of the ANOVA (see Table 2) showed a significant main effect of teaching approach on English proficiency, *F*(1,116) = 29.41, *p* < 0.001, *η_p_*^2^ = 0.20. Based on Cohen’s (1988) benchmarks, the effect size was large. It indicated that teaching approach explained 20% of the variance in the dependent variable. The pre-service teachers in the FC (*M* = 64.80, SD = 6.97) scored significantly higher on the test than those in the TT (*M* = 58.10, SD = 7.65). Besides, there was a significant main effect of educational background on English proficiency, *F*(1,116) = 12.02, *p =* 0.001, *η_p_*^2^ = 0.09. Educational background accounted for 9% of the variance in the English proficiency. The urban pre-service teachers (*M* = 63.59, SD = 8.64) scored significantly higher than the rural ones (*M* = 59.31, SD = 6.77). Additionally, there was a significant interaction effect between teaching approach and educational background on the English proficiency, *F*(1,116) = 9.88, *p* = 0.002, *η_p_*^2^ = 0.08, indicating that the effect of educational background on English proficiency were different in the flipped and traditional groups. The interaction explained 8% of the variance.

**Table 2:** Tests of between-subjects effects.

Dependent variable: IELTS post-test						
Source	Type III sum of squares	Degree of freedom	Mean square	*F*	Sig.	Partial η^2^
Corrected model	2349.52^a^	3	783.17	17.11	0.000	0.31
Intercept	453132.30	1	453132.30	9896.96	0.000	0.99
TA	1346.70	1	1346.70	29.41	0.000	0.20
EB	550.41	1	550.41	12.02	0.001	0.09
TA * EB	452.41	1	452.41	9.88	0.002	0.08
Error	5311.06	116	45.79			
Total	460792.88	120				
Corrected total	7660.58	119				

a *R*^2^ = 0.307 (Adjusted *R*^2^ = 0.289).

**Figure 1:**
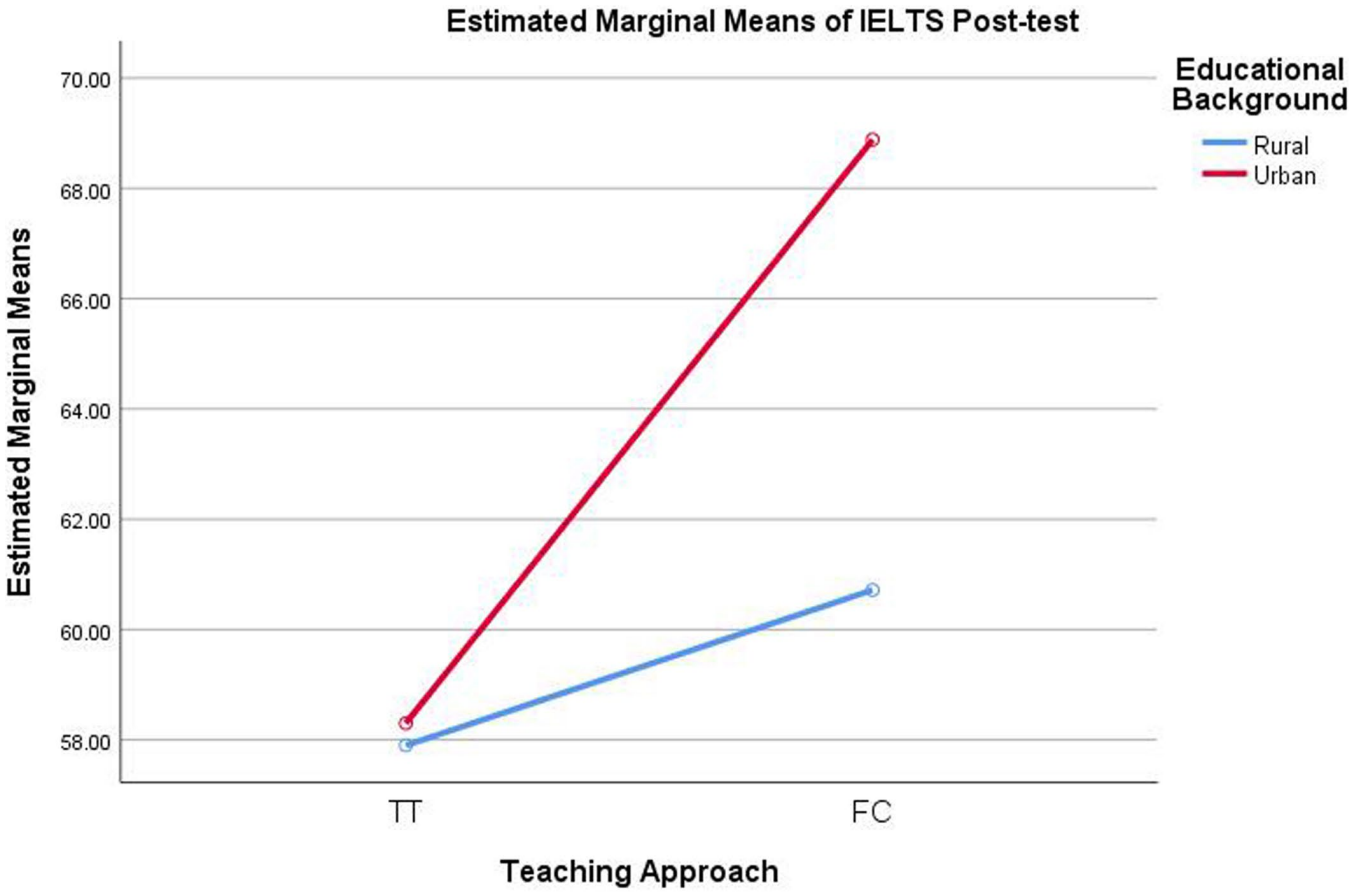
Interaction plot for IELTS post-test.

Since the interaction was significant (Figure 1), simple effect analysis was performed. Referring to the pairwise differences based on estimated marginal means, urban pre-service teachers in the FC achieved significantly higher English proficiency than their urban peers in the TT (MD = 10.58, *p* < 0.001). For rural pre-service teachers, no significant difference was observed between the two groups (MD = 2.82, *p* = 0.110). In the FC, the urban pre-service teachers significantly outperformed their rural classmates (MD = 8.17, *p* < 0.001). However, in the TT, no significant difference was found in the English proficiency of urban and rural pre-service teachers (MD = 0.40, *p* = 0.819). Overall, the pre-service teachers who received the combined effect of flipped classroom and urban educational background presented substantially higher English proficiency than those under other conditions.

## 5 Discussion

This study aimed to investigate the impact of flipped classroom on English proficiency of first-year Chinese pre-service teachers. Additionally, it sought to determine whether educational background interacted with teaching approach in affecting the language proficiency of the learners. In an attempt to reach the research objectives, the quantitative data were gathered with the IELTS tests. The results indicated that there was a statistically significant difference in English proficiency among the pre-service teachers, with the flipped group outperforming the traditional group. The first null hypothesis was rejected. The results also revealed a significant interaction between educational background and teaching approach in terms of the pre-service teachers’ English proficiency. The second null hypothesis was rejected.

Contrary to the first null hypothesis, the study findings supported the positive impact of the flipped classroom on English proficiency, which was consistent with some previous studies (Arslan, 2020; He, 2020; Turan and Akdag-Cimen, 2020; Kusuma, 2022). The significant difference between the two groups in terms of English proficiency could be attributed to various factors. The higher English proficiency of the pre-service teachers in the flipped group may be due to more opportunities for in-depth processing, frequent practice and reinforcement of higher-level cognitive skills, as well as the collaborative, scaffolding, and constructivist flipped learning environment. In contrast, the lower level of English proficiency of those in the traditional group might be attributable to the passive learning style among the Chinese EFL learners in the teacher-centered classroom environment.

To be specific, the flipped classroom may support the pre-service teachers to progressively achieve higher levels in Bloom’s taxonomy, starting with lower-order cognitive tasks before class and then advancing to more complex cognitive tasks during class hours. The higher-level cognitive activities in flipped classes, as suggested by Djamàa (2020), could facilitate the integration of knowledge and prompt corrective actions, resulting in higher English proficiency levels for the EFL learners. Moreover, the flipped classroom, which aligns with socio-constructivist theories, emphasized the active construction of knowledge through social interactions and authentic learning environments. By engaging in communicative and collaborative tasks, the pre-service teachers could actively build their own knowledge, scaffolded and mediated by their peers and instructors. It was very likely that the communicative, collaborative, and active learning atmosphere extended numerous opportunities outside and inside the classroom for the pre-service teachers to have linguistically rich interactions with learning materials, peers, and the instructor, resulting in significantly enhanced English proficiency levels. In contrast, the traditional classroom might intensify the passivity of the Chinese EFL learners influenced by the traditional Confucian culture. As remarked by Karjanto and Simon (2019), many East Asian students who grow up in the Confucian Heritage Culture are often portrayed as quiet and passive learners and may be accustomed to passively listening to their teachers’ instructions, leading to reticence (Chang and Lin, 2019) and a lack of participation in class activities (Suranakkharin, 2017; Lee and Wallace, 2018; Karjanto, 2021). These typical learning features, which might exacerbate in the traditional teacher-centered classroom, probably hindered the social construction of language knowledge and development of associated skills of the pre-service teachers, leading to lower English proficiency levels compared to their counterparts.

Furthermore, as opposed to the second null hypothesis, the results revealed a significant interaction effect between educational background and teaching approach, indicating that the impact of educational background on English proficiency differed in the flipped and traditional groups. Specifically, in the flipped classroom, urban pre-service teachers achieved higher English proficiency than their rural counterparts, whereas no significant difference was found in the traditional classroom. Additionally, the urban pre-service teachers in the flipped classroom outperformed their urban peers in the traditional classroom, while the rural pre-service teachers did not differ significantly in the two classes.

The finding concerning the higher English proficiency of urban pre-service teachers when compared with their rural classmates in the flipped classroom corroborated previous research by Lv and Wang (2016) that reported polarized achievements of urban and rural students in flipped language classes. These researchers argued that the flipped classroom may bring more positive learning outcomes to urban students than rural ones, since those from rural areas may lack in sufficient learner autonomy and information technology capacities to quickly adapt to the new teaching model. This study supported this explanation, as the significant difference in English proficiency between urban and rural pre-service teachers may be attributed to their different adaptability levels in the flipped learning environment. As critically commented by Adnan (2017), technology in education can be a “gift” or a “curse” for students, depending on their capacities to utilize digital tools and resources. As a technology-embedded teaching approach, the flipped classroom may favor urban pre-service teachers, who may possess higher digital abilities to utilize technology devices, and disadvantage rural pre-service teachers. The latter might face significant challenges not only in self-paced learning prior to class but also in collaborative social interactions during technology-aided classes. Compared to their rural peers, urban pre-service teachers may better integrate the two key components of the flipped classroom (preparatory work outside of class and interactive activities inside the classroom) with more skillful use of technological tools. Therefore, they may more easily and quickly adjust to and benefit from their flipped learning, ultimately achieving higher English proficiency.

Moreover, the study found no significant difference between the English proficiency of urban and rural pre-service teachers in the traditional classroom. This new finding was contrary to the results of previous studies that reported significant achievement gaps between Chinese urban and rural EFL learners in conventional language classrooms due to economic and educational disparities between urban and rural areas (Gao et al., 2019; Liu and Wang, 2021). In this study, although urban pre-service teachers performed slightly better than their rural counterparts in the teacher-centered environment, there was no conspicuous gap in their English proficiency. This may be due to the strong influence of traditional Confucian and Taoist culture on both urban and rural pre-service teachers, causing them to feel hesitant and anxious while expressing themselves and communicating with their peers, and to show deference to their teacher’s authority. The high affective filter of these learners may have resulted in limited improvement in English proficiency, regardless of their educational backgrounds. These findings suggested that the traditional teaching approach, which emphasized the authoritative and central role of the instructor, may not be an effective instructional approach for stimulating the enhancement of English proficiency for both urban and rural pre-service teachers in this context.

Furthermore, the study revealed higher English proficiency of the urban pre-service teachers in the flipped group than their urban peers in the traditional group. This intriguing finding might be attributed to greater opportunities afforded by the flipped approach for the urban learners to utilize their strengths in technology and digital literacy. As digital natives (Prensky, 2001), these urban pre-service teachers might have been well-resourced with digital devices and grown up with a relatively high level of technical proficiency. Therefore, it was likely that they could benefit from the frequent use of digital devices in the flipped classroom to deepen their understanding of key concepts prior to class and engage in advanced cognitive development via active social interactions with their peers and instructor. In contrast, the urban pre-service teachers in the traditional classroom had fewer chances to employ their digital skills and tools to support their learning, as most of the class time was devoted to teacher-centered instruction and content recall, which limited their cognitive development. Further, they were left to tackle complex cognitive tasks independently after class, with less support and scaffolding available to their peers in the interactive and technology-aided flipped classroom. Overall, these findings suggested that the flipped classroom seemed to be a promising approach to enhance the English proficiency of urban pre-service teachers by leveraging technology and their digital capacities to facilitate advanced cognitive development.

Additionally, the new finding about the insignificant difference of the rural pre-service teachers in the two groups might be due to the inadequacy of learner autonomy of the rural pre-service teachers in the flipped classroom. While the utilization of digital tools and technology-assisted flipped teaching benefited the urban pre-service teachers in their study, it may not have resulted in the same outcomes for their rural peers. Even if offered a good number of opportunities to direct their learning in and out of the flipped classroom, the rural pre-service teachers, who might have fewer digital skills due to their economic constraints (Liu and Wang, 2021; Zhang, 2022), may encounter multiple challenges to handle their flipped learning with necessary autonomous abilities. As noted by Shahid et al. (2020), the lack of learner autonomy can be a significant hurdle impeding the development of EFL learners’ English proficiency. Thus, it was not surprising that the rural pre-service teachers in the flipped classroom who might be deficient in their learner autonomy could not achieve significantly higher English proficiency than their rural peers in the traditional classroom. This finding highlighted the great need to provide adequate support to Chinese rural pre-service teachers to help them maximize the potential benefits of the flipped classroom.

## 6 Conclusion

The present study made a significant contribution to the literature by examining the impact of the flipped classroom on English proficiency of first-year pre-service teachers in China with urban and rural educational backgrounds. The findings suggested that the flipped classroom approach was more effective in improving the English proficiency of the pre-service teachers, especially those with an urban educational background. However, the results also indicated that rural pre-service teachers may need additional support to gain equal benefits from this approach. To address this issue, teacher educators should provide sufficient training opportunities for flipped classroom, allow for an extended period of time for practice and adaptation of flipped learning and the use of technological tools, gradually delegate learning responsibilities to the pre-service teachers, and take on a supportive role in guiding the learners, particularly those with rural educational backgrounds, in their flipped language learning process. Additionally, it is recommended that educational institutions and authorities in the context provide adequate administrative, financial, and technical support to encourage the implementation of the flipped classroom, such as providing flipped classroom and technology training to both teachers and students, promoting staff collaboration and communication, increasing investment to teacher educational institutions to improve ICT infrastructure, and initiating programs to promote technology-assisted EFL education in rural secondary schools.

The current study had several limitations that warrant acknowledgement. First, the experiment’s duration was relatively short, and the sample size was small, as the participants were recruited from a single university. Second, the research instrument was limited, resulting in an insufficient amount of data collected. To address these limitations, future research could benefit from conducting longitudinal studies to examine the long-term effects of the flipped classroom on pre-service teachers’ English proficiency. Additionally, delving into the cultivation of key competencies crucial for sustainability of the learners, such as critical thinking, problem-solving, and collaboration, as highlighted by Karjanto and Acelajado (2022), would be beneficial. Expanding the sample size and scope of the English courses could lead to more generalizable results. Employing a variety of instruments to collect both quantitative and qualitative data would enhance the study’s comprehensiveness. Investigating different factors, such as technology abilities, influencing the outcomes would offer a deeper understanding of the subject matter. Further, considering possible gender differences in learning within the flipped classroom, it is recommended to take into account gender characteristics when applying and further improving the pedagogy, potentially through algorithm-based machine learning. Lastly, probing into specific challenges encountered by pre-service teachers in flipped English classes, especially within Confucian Heritage Culture contexts, holds promise. Identifying these hurdles and proposing targeted solutions could pave the way for more effective and culturally sensitive pedagogical strategies.

Despite these limitations, this study contributed valuable empirical evidence and practical insights into the effectiveness of the flipped classroom approach in enhancing the English proficiency of first-year Chinese pre-service teachers. The findings of this study established a solid groundwork for subsequent research in China and in educational contexts with similar characteristics.

## Data availability statement

The datasets generated for this study are available on reasonable request to the corresponding author.

## Ethics statement

The study involving humans was approved by the Human Ethics Committee, Taylor’s University. The study was conducted in accordance with the local legislation and institutional requirements. The participants provided their written informed consent to participate in this study.

## Author contributions

WC: Conceptualization, Formal analysis, Investigation, Methodology, Resources, Validation, Writing – original draft, Writing – review & editing.

## References

[ref1] Abdullah M. Y. Hussin S. Ismail K. (2019). Investigating the effects of the flipped classroom model on Omani EFL learners’ motivation level in English speaking performance. Educ. Inf. Technol. 24, 2975–2995. doi: 10.1007/S10639-019-09911-5

[ref2] Adnan M. (2017). Perceptions of senior-year ELT students for flipped classroom: a materials development course. Comput. Assist. Lang. Learn. 30, 204–222. doi: 10.1080/09588221.2017.1301958

[ref3] Alhamami M. Costello H. (2019). Pre-service EFL teachers’ expectations, needs, and challenges in a language learning and technology course. J. Lang. Teach. Res. 10, 593–602. doi: 10.17507/JLTR.1003.23

[ref4] Alhamami M. Khan M. R. (2019). Effectiveness of flipped language learning classrooms and students’ perspectives. J. English Foreign Lang. 9, 71–86. doi: 10.23971/JEFL.V9I1.1046

[ref5] Altas E. A. Enisa M. (2021). The impact of flipped classroom approach on the writing achievement and self-regulated learning of pre-service English teachers. Turk. Online J. Distance Educ. 22, 66–88. doi: 10.17718/TOJDE.849885

[ref6] Arslan A. (2020). A systematic review on flipped learning in teaching English as a foreign or second language. J. Lang. Linguist. Stud. 16, 775–797. doi: 10.17263/JLLS.759300

[ref7] Basal A. (2015). The implementation of a flipped classroom in foreign language teaching. Turk. Online J. Distance Educ., 28–37. doi: 10.17718/TOJDE.72185

[ref8] Bergmann J. Sams A. (2012a). Before you flip, consider this. Phi Delta Kappan 94:25. doi: 10.1177/003172171209400206

[ref9] Bergmann J. Sams A. (2012b). Flip your classroom: Reach every student in every class every day. Washington, DC: International Society for Technology in Education.

[ref10] Bishop J. Verleger M. A. (2013). “The flipped classroom: a survey of the research” in 2013 ASEE Annual Conference & Exposition. 23.1200.1201–1223.1200.1218

[ref11] Bloom B. S. (1984). Taxonomy of educational objectives. London: Pearson.

[ref12] Cabi E. (2018). The impact of the flipped classroom model on students' academic achievement. Int. Rev. Res. Open Distance Learn. 19, 202–221. doi: 10.19173/IRRODL.V19I3.3482

[ref13] Cambridge E. S. O. L. (2019). Cambridge IELTS 9 student's book with answers: Authentic examination papers from Cambridge ESOL. Cambridge: Cambridge University Press.

[ref14] Chang C. Lin H. C. K. (2019). Classroom interaction and learning anxiety in the IRS-integrated flipped language classrooms. Asia-Pacific Educ. Res. 28, 193–201. doi: 10.1007/S40299-018-0426-X

[ref15] Chen W. (2019). Fanzhuan ketang moshixia yingyu zhuanye xuesheng yingyu xuexi jiaolv de diaocha yu yanjiu [A survey of English learning anxiety of English majors under flipped classroom]. Chifeng xueyuan xuebao 40, 124–129.

[ref16] Chen Z. (2020). A contrastive study on the causes of foreign language classroom anxiety of high school students in urban and rural areas. Master's thesis, Hainan Normal University.

[ref17] Chinese Ministry of Education (2012). Plan for ICT in Chinese Ministry of Education 2011–2020. Available at: http://old.moe.gov.cn/publicfiles/business/htmlfiles/moe/s3342/201203/xxgk_133322.html (Accessed November 30, 2023).

[ref18] Cohen J. (1988). Statistical power analysis for the behavioral sciences. Hillsdale, NJ: Lawrence Erlbaum Associates.

[ref19] Communist Party of China Central Committee and The State Council (2018). National rural revitalization plan (2018–2022). Available at: http://www.gov.cn/zhengce/2018-09/26/content_5325534.htm (Accessed November 30, 2023).

[ref20] Cuong P. H. (2021). English language education in rural areas: current issues, complexities and ways forward. VNU J. Sci. Educ. Res. 37, 39–48. doi: 10.25073/2588-1159/VNUER.4538

[ref21] Deepa R. (2021). A study on rural and urban learners in learning English language and their difficulties in sentence formation in English. Int. J. Multidiscip. Educ. Res. 10, 105–110.

[ref22] Djamàa S. (2020). Lecture in the living room, homework in the classroom: the effects of flipped instruction on graduate EFL students’ exam performance. Comput. Sch. 37, 141–167. doi: 10.1080/07380569.2020.1795513

[ref23] Eppard J. Rochdi A. (2017). “A framework for flipped learning” in International Association for Development of the information society (IADIS) international conference on mobile learning (International Association for Development of the Information Society).

[ref24] Evans W. Savage J. (2015). Developing a local curriculum: Using your locality to inspire teaching and learning. London: Routledge.

[ref25] Fazel I. Ahmadi A. (2011). On the relationship between writing proficiency and instrumental/integrative motivation among Iranian IELTS candidates. Theory Pract. Lang. Stud. 1, 747–757. doi: 10.4304/tpls.1.7.747-757

[ref26] Feng W. Liu Y. Qu L. (2019). Effect of land-centered urbanization on rural development: a regional analysis in China. Land Use Policy 87:e104072. doi: 10.1016/j.landusepol.2019.104072

[ref27] Gao Y. Li R. Li S. Wang M. G. (2019). Nongcun zhongxue yingyu jiaoyu weiguan shengtai shizheng yanjiu [An empirical study on English education in rural areas from educational micro-ecosystem perspective]. Donghua ligong daxue xuebao 38, 62–65.

[ref28] García-Sánchez S. Santos-Espino J. M. (2017). Empowering pre-service teachers to produce ubiquitous flipped classes. PROFILE Issues Teach. Prof. Dev. 19, 169–185. doi: 10.15446/PROFILE.V19N1.53857

[ref29] Getie A. S. (2020). Factors affecting the attitudes of students towards learning English as a foreign language. Cogent Educ. 7:e1738184. doi: 10.1080/2331186X.2020.1738184

[ref30] Ghufron M. A. Nurdianingsih F. (2019). Flipped teaching with call in EFL writing class: how does it work and affect learner autonomy? Eur. J. Educ. Res. 8, 983–997. doi: 10.12973/EU-JER.8.4.983

[ref31] Glassdoor Team (2021). Answering questions about educational background. Available at: https://www.glassdoor.com/blog/guide/educational-background/ (Accessed November 30, 2023).

[ref32] Gok D. Bozoglan H. Bozoglan B. (2021). Effects of online flipped classroom on foreign language classroom anxiety and reading anxiety. Comput. Assist. Lang. Learn. 36, 840–860. doi: 10.1080/09588221.2021.1950191

[ref33] Haghi M. (2021). The effective integration of flipped classroom in ELT contexts: a review of recent literature. Arab World English J. 2, 120–132. doi: 10.24093/AWEJ/MEC2.9

[ref34] Hashemi A. Daneshfar S. (2018). A review of the IELTS test: focus on validity, reliability, and washback. Indonesian J. English Lang. Teach. Appl. Linguist. 3, 39–52. doi: 10.21093/ijeltal.v3i1.123

[ref35] He J. (2020). Research and practice of flipped classroom teaching mode based on guidance case. Educ. Inf. Technol. 25, 2337–2352. doi: 10.1007/S10639-020-10137-Z

[ref36] Ingram D. Bayliss A. (2007). IELTS as a predictor of academic language performance (part I). IELTS Res. Rep. 7, 1–68,

[ref37] Jenkins J. (2003). World Englishes. London: Routledge.

[ref38] Jiang H. X. (2019). Xinshidai waiyu jiaoyu gaige de jidian gouxiang [Some thoughts on reform of foreign language education in the new era]. Waiyujie 1, 13–16.

[ref39] Karaaslan H. Çelebi H. (2017). ELT teacher education flipped classroom: an analysis of task challenge and student teachers’ views and expectations. J. Lang. Linguist. Stud. 13, 643–666,

[ref40] Karjanto N. (2021). “Active participation and student journal in Confucian heritage culture mathematics classrooms” in Proceedings of the international conference on mathematics, geometry, statistics, and computation (IC-MaGeStiC 2021) (Dordrecht: Atlantis Press), 89–91.

[ref41] Karjanto N. Acelajado M. J. (2022). Sustainable learning, cognitive gains, and improved attitudes in college algebra flipped classrooms. Sustain. For. 14:12500. doi: 10.3390/su141912500

[ref42] Karjanto N. Simon L. (2019). English-medium instruction calculus in Confucian-heritage culture: flipping the class or overriding the culture? Stud. Educ. Eval. 63, 122–135. doi: 10.1016/J.STUEDUC.2019.07.002

[ref43] Ke Z. (2016). Kecheng gaige yu nongcun xuesheng de xueye chenggong jihui- Jiyu A’shi banian zhongkao shuju de fenxi [Curriculum reform and the opportunity of academic success of rural students - Based on eight-year data of senior high school entrance examination from city A]. Jiaoyu yanjiu, 10, 95–105,

[ref44] Kırmızıa O. Kömeç F. (2019). The impact of the FC on receptive and productive vocabulary learning. J. Lang. Linguist. Stud. 15, 437–449. doi: 10.17263/jlls.586096

[ref45] Koo T. K. Li M. Y. (2016). A guideline of selecting and reporting intraclass correlation coefficients for reliability research. J. Chiropr. Med. 15, 155–163. doi: 10.1016/J.JCM.2016.02.012, PMID: 27330520 PMC4913118

[ref46] Kurt G. (2017). Implementing the flipped classroom in teacher education: evidence from Turkey. Educ. Technol. Soc. 20, 211–221.

[ref47] Kusuma I. P. I. (2022). How does a TPACK-related program support EFL pre-service teachers’ flipped classrooms? Learn. J. Lang. Educ. Acquis. Res. Netw. 15, 300–325.

[ref48] Lage M. J. Platt G. J. Treglia M. (2000). Inverting the classroom: a gateway to creating an inclusive learning environment. J. Econ. Educ. 31, 30–43. doi: 10.1080/00220480009596759

[ref49] Lakarnchua O. Balme S. Matthews A. (2020). Insights from the implementation of a flipped classroom approach with the use of a commercial learning management system. Turk. Online J. Distance Educ. 21, 63–76. doi: 10.17718/TOJDE.762027

[ref51] Lee G. Wallace A. (2018). Flipped learning in the English as a foreign language classroom: outcomes and perceptions. TESOL Q. 52, 62–84. doi: 10.1002/TESQ.372

[ref52] Li G. Jee Y. Sun Z. (2018). Technology as an educational equalizer for EFL learning in rural China? Evidence from the impact of technology-assisted practices on teacher-student interaction in primary classrooms. Lang. Lit. 20, 159–184. doi: 10.20360/LANGANDLIT29415

[ref53] Liu J. L. (2021). Zai nongcun chuzhong yingyu jiaoxue zhong tuijin fanzhuan ketang de shijian yanjiu [Practical research on promoting flipped classroom in rural junior middle school English teaching]. Jiazhang 21, 122–123.

[ref54] Liu X. Gu J. Xu J. (2023). The impact of the design thinking model on pre-service teachers’ creativity self-efficacy, inventive problem-solving skills, and technology-related motivation. Int. J. Technol. Des. Educ. 34, 167–190. doi: 10.1007/S10798-023-09809-X

[ref55] Liu Y. Wang H. (2021). English education in rural secondary schools in Southwest China: problems and countermeasures under the rural vitalization strategy. Int. J. English Lang. Teach. 8, 32–40. doi: 10.5430/IJELT.V8N2P32

[ref56] Loucky J. P. (2017). "Studies of flipping classes with Asian students," in Flipped instruction methods and digital technologies in the language learning classroom, eds. Loucky J. P. and Ware J. L. Hershey, Penn: IGI Global, 64–90.

[ref57] Lv T. T. Wang N. (2016). Jiyu SPOC+ shuzihua jiaoxue ziyuan pingtai de fanzhuan ketang jiaoxue moshi yanjiu- Yi daxue yingyu weili [A study on the establishment and effect of the flipped classroom mode for SPOC+ teaching resource platform as applied in college English teaching]. Zhongguo dianhua jiaoyu 5, 85–90.

[ref58] National Bureau of Statistics (2020). Announcement on the renewal of the national statistical zoning code and urban and rural zoning code. Available at: http://www.stats.gov.cn/tjsj/tjbz/tjyqhdmhcxhfdm/2020/index.html (Accessed November 30, 2023).

[ref59] Noor H. A. (2020). A probe into the different aspects of ‘validity’ and ‘reliability’ of IELTS writing test. Int. J. English Literat. Soc. Sci. 5, 968–972. doi: 10.22161/ijels.54.21

[ref60] Ömer Ö. Akçayoğlu D. İ. (2021). Examining the roles of self-efficacy beliefs, self-regulated learning and foreign language anxiety in the academic achievement of tertiary EFL learners. Particip. Educ. Res. 8, 357–372. doi: 10.17275/per.21.43.8.2

[ref61] Ou C. (2017). A review on language learner autonomy research in China (2006-2016): based on 12 key domestic journals. English Lang. Teach. 10, 76–86. doi: 10.5539/ELT.V10N11P76

[ref62] Pang Y. (2022). The role of web-based flipped learning in EFL learners’ critical thinking and learner engagement. Front. Psychol. 13:1008257. doi: 10.3389/FPSYG.2022.1008257, PMID: 36405154 PMC9667938

[ref63] Pariyanto P. Pradipta B. (2020). Factors influencing an EFL learner’s proficiency: an English teacher’s perspective. J. Lang. Lit. Cult. Stud. 2, 89–97. doi: 10.30996/ANAPHORA.V2I2.3369

[ref64] Prensky M. (2001). Digital natives, digital immigrants part 1. Horizon 9, 1–6. doi: 10.1108/10748120110424816

[ref65] Qu P. (2017). Jiyu weixin pingtai de zonghe yingyu kouyu fanzhuan ketang moshi tanjiu [A flipped classroom model of oral practice in integrated English teaching based on WeChat platform]. Ningbo Jiaoyu xueyuan xuebao 17, 23–26.

[ref66] Quaid E. D. (2018). Reviewing the IELTS speaking test in East Asia: theoretical and practice-based insights. Lang. Test. Asia 8, 1–9. doi: 10.1186/s40468-018-0056-5

[ref67] Rajabi P. Mahmoodi K. Hosseini S. A. (2021). Flipped classroom model and its impact on Iranian EFL learners’ classroom anxiety and listening performance. Comput. Lang. Learn. Electron. J. 22, 1–16.

[ref68] Roth C. Suppasetseree S. (2016). “Flipped classroom: can it enhance English listening comprehension for pre-university students in Cambodia” in Proceedings of classic: Learning in and beyond the classroom: Ubiquity in foreign language education, 255–264.

[ref69] Rural Education Action Program (2018). Computers as tutors: Leveraging PCs to advance learning in China’s rural schools. Available at: https://reap.fsi.stanford.edu/sites/default/files/REAP116-EN.pdf (Accessed November 30, 2023).

[ref70] Shahid C. Ong E. T. Wong K. T. Perveen A. (2020). Expectations and reality of learner autonomy and communicative competence in Pakistani higher education institutions: a review. Int. J. Educ. Psychol. Couns. 5, 91–101. doi: 10.35631/IJEPC.534007

[ref71] Strelan P. Osborn A. Palmer E. (2020). The flipped classroom: a meta-analysis of effects on student performance across disciplines and education levels. Educ. Res. Rev. 30:e100314:100314. doi: 10.1016/j.edurev.2020.100314

[ref72] Sukerti G. N. A. Rudiastari E. Susana K. Y. (2020). The effectiveness of flipped learning in teaching writing. Soshum J. Sos. dan Hum. 10, 78–92. doi: 10.31940/soshum.v10i1.1634

[ref73] Suranakkharin T. (2017). Using the flipped model to foster Thai learners' second language collocation knowledge. 3L Lang. Linguist. Lit. 23, 1–20. doi: 10.17576/3L-2017-2303-01

[ref74] Talbert R. (2012). Inverted classroom. Colleagues 9, 89–95.

[ref75] Turan Z. Akdag-Cimen B. (2020). Flipped classroom in English language teaching: a systematic review. Comput. Assist. Lang. Learn. 33, 590–606. doi: 10.1080/09588221.2019.1584117

[ref76] University of Cambridge Local Examinations Syndicate (2007). IELTS handbook 2007. Cambridge: University of Cambridge Local Examinations Syndicate.

[ref001] Wang H. Mansor N. S. Wang X. Hoon L. Darmi R. (2021). Application of flipped classroom for English education based on network information technology. J. Balk. Tribol. Assoc. 27, 321–331.

[ref77] Wang Y. (2020). A study on college English high-efficiency class based on blended teaching mode of flipped classroom. Theory Pract. Lang. Stud. 10, 1066–1071. doi: 10.17507/tpls.1009.08

[ref78] Wen Q. F. (2019). Xinzhongguo waiyu jiaoyu 70 nian: Chengjiu yu tiaozhan [Foreign language education in China in the past 70 years: Achievements and challenges]. Waiyu jiaoxue yu yanjiu 5, 735–745.

[ref79] Wu S. Xu Y. Han J. Jiang M. (2021). Detecting the factors affecting the learning performance of students with different learning styles in flipped learning. ICIC Exp. Lett. Part B Appl. 12, 1137–1144.

[ref80] Wu X. C. Zhang H. M. (2021). Fangyi qijian shiping xianshang ketang dui xuesheng xuexi xingqu yingxiang de ge’an yanjiu- Yi yingyu zhuanye zonghe yingyu weili [The influence of online classroom teaching model on students’ satisfaction and learning interests under the epidemic COVID-19: a case of integrated English course]. Zhongguo duomeiti yu wangluo jiaoxue xuebao 3, 51–54.

[ref81] Xiu G. (2021). Xinshidai waiyu zhuanye jiaoyu de xinzhuanbian [Changes and innovation in the education of English majors in the new era]. Zhongguo waiyu 1, 21–22.

[ref82] Yang C. C. R. (2017). An investigation of the use of the ‘flipped classroom’ pedagogy in secondary English language classrooms. J. Inf. Technol. Educ. Innov. Pract. 16, 1–20. doi: 10.28945/3635

[ref83] Yang C. C. R. Chen Y. (2020). Implementing the flipped classroom approach in primary English classrooms in China. Educ. Inf. Technol. 25, 1217–1235. doi: 10.1007/s10639-019-10012-6

[ref84] Yen D. Kuzma J. (2009). Higher IELTS score, higher academic performance? The validity of IELTS in predicting the academic performance of Chinese students. Worcester J. Learn. Teach. 3, 1–7.

[ref85] Yue A. Tang B. Shi Y. Tang J. Shang G. Medina A.. (2018). Rural education across China's 40 years of reform: past successes and future challenges. China Agric. Econ. Rev. 10, 93–118. doi: 10.1108/CAER-11-2017-0222

[ref86] Zhang S. (2022). Chengshi, xiancheng ji nongcun xiaoxuesheng yingyu xuexi qinggan duibi yanjiu [A comparative study of English learning emotions of primary school students in cities, counties and rural areas]. Jiaoxue yu guanli 12, 27–32.

[ref87] Zhang J. Q. Guo L. (2018). Benkesheng ketang jiaolv yanjiu: Waiyu ketang jiaolv liangbiao (FLCAS) de xinxiaodu zaijianyan [Study on language anxiety of non-English majors: An examination of FLCAS’s reliability and construct validity]. Huabei ligong daxue xuebao 18, 105–112,

[ref88] Zhang H. Li J. Jiao L. Ma W. Guan C. (2016). The adjustment and effects of vocabulary teaching strategies in flipped classroom. Creat. Educ. 7, 1966–1973. doi: 10.4236/ce.2016.714199

